# Adherence-based experiences with a personalized self-care program for type 2 diabetes in South Korea: a mixed-methods study

**DOI:** 10.7586/jkbns.25.067

**Published:** 2025-11-26

**Authors:** Haejung Lee, DaeEun Lee, Mihwan Kim

**Affiliations:** 1 College of Nursing, Pusan National University, Yangsan, Korea; 2 Research Institute of Nursing Science, Pusan National University, Yangsan, Korea

**Keywords:** Diabetes mellitus, Mobile applications, Qualitative research, Self-management

## Abstract

**Purpose:**

This study aimed to explore the experiences of patients with type 2 diabetes (T2D) according to their adherence to a personalized self-care (PSC) program and to evaluate its effects on self-care activities and hemoglobin A1c (HbA1c) levels 18 months after baseline.

**Methods:**

A convergent mixed-methods design was employed. Qualitative interviews were conducted with 33 participants who demonstrated high adherence (adherence group, AG) and 17 participants who demonstrated low adherence (non-adherence group, NAG). Data were analyzed using Elo and Kyngäs’s content analysis approach. The program’s effects were evaluated by comparing the mean differences between pre-test and 18-month follow-up data using SPSS version 23.0.

**Results:**

Participants in the AG used the app for self-monitoring, which promoted self-reflection and improved diabetes self-care. Participants in the NAG expressed an intentions to improve but reported low motivation due to limited willpower, busy schedules, and insufficient awareness of the diabetes severity. Foot care significantly improved both within (Z = −3.98, *p* < .001) and between groups (t = −2.22, *p* = .031), with the most notable improvement observed in the AG. Significant improvements in general diet (t = −2.68, *p* = .012) and HbA1c levels (Z = −2.93, *p* = .003) were observed only in the AG.

**Conclusion:**

The finding suggested that PSC program enhanced diabetes self-care activities, particularly in the areas of general diet, foot care, and HbA1c control. To enhance self-care adherence among individuals with T2D, mobile apps should offer goal setting, progress tracking, simplified data entry, and reminders.

## INTRODUCTION

The global prevalence of type 2 diabetes (T2D) is increasing rapidly, primarily because of dietary changes and reduced physical activity associated with industrialization and urbanization. According to the World Health Organization [1], 14.0% of adults aged 18 years and older were living with diabetes in 2022. By 2050, the number of individuals with T2D is projected to reach between 657 million and 1.095 billion, depending on future prevalence trends [2]. A previous study that analyzed cardiovascular disease (CVD) risk based on comorbidities among adults aged 30 to 90 years with T2D reported that having T2D for 5 years or longer was associated with a 4.17~12.04-fold increase in CVD risk. Furthermore, the coexistence of hypertension or chronic kidney disease with T2D substantially increased the risk of myocardial infarction and stroke [3]. These findings highlight the critical importance of effective self-management in preventing and managing complications, particularly among middle-aged and older adults with T2D.

Effective diabetes self-management involves medication adherence, dietary regulation, physical activity, weight control, blood glucose monitoring, and smoking cessation [4]. The use of mobile applications for T2D self-management has increased in recent years and has demonstrated clinically meaningful outcomes. For example, a mobile app that provided personalized goals and automated feedback improved self-efficacy, diabetes self-care behaviors, and vegetable intake, while reducing hemoglobin A1c (HbA1c) and cholesterol levels [5]. The integration of educational materials, motivational interviewing, and personalized feedback on glycemic control has also been shown to be effective in diabetes management [6]. In another study, participants who received individualized calorie intake goals based on their height, weight, and physical activity level through a mobile app—and who recorded their dietary intake and weight over 8-week period while receiving educational support—experienced significant weight loss [7].

Despite the documented benefits of mobile app–based interventions, high dropout rates remain a significant challenge. A meta-analysis by Meyerowitz-Katz et al. [8] reported an average attrition rate of 43.0% for mobile interventions targeting chronic conditions. For example, Zimmermann et al. [6] observed that participants with low app engagement showed no significant improvement in HbA1c levels. Similarly, in a 3-month mobile intervention for patients with T2D or hypertension, Oh et al. [9] reported low entry rates for dietary intake and physical activity (24.9% and 5.3%, respectively) and no significant improvements in weight, body mass index (BMI), or blood pressure. However, adherence to medication entry was relatively high (84.0%), and increased medication tracking was significantly associated with improved HbA1c levels [9]. These findings suggest that adherence to mobile app–based interventions is a critical factor influencing health outcomes.

Previous studies have identified several barriers to participation in mobile app–based interventions for diabetes self-management, including difficulties using the app, limited awareness of its benefits, busy daily schedules, and low perceived disease severity [10-12]. However, these studies primarily focused on comparisons between app users and non-users [11], intervention groups alone [10], or individuals who dropped out during the intervention [12]. Furthermore, few studies [6,8,9] have examined both quantitative outcomes and qualitative experiences across groups with varying adherence levels. Given the critical role of adherence in shaping health outcomes, a comparative analysis of the experiences and outcomes of the adherence group (AG) and non-adherence group (NAG) is essential for developing effective strategies to enhance engagement and maximize the impact of mobile health interventions.

This study aimed to explore participants' experiences with the personalized self-care (PSC) program and to evaluate its effects on health outcomes according to their adherence levels. The effectiveness of the program was evaluated by assessing changes in self-care self-efficacy, diabetes self-management, and HbA1c over time and between the AG and NAG.

## METHODS

### 1. Study design

This study employed a convergent mixed-methods design to explore experiences with the PSC program and to evaluate its effects on health outcomes based on adherence levels among individuals with T2D. This design integrated qualitative and quantitative data to provide a comprehensive understanding of the application’s effectiveness in T2D self-management [13].

### 2. Participants

**Figure 1.**
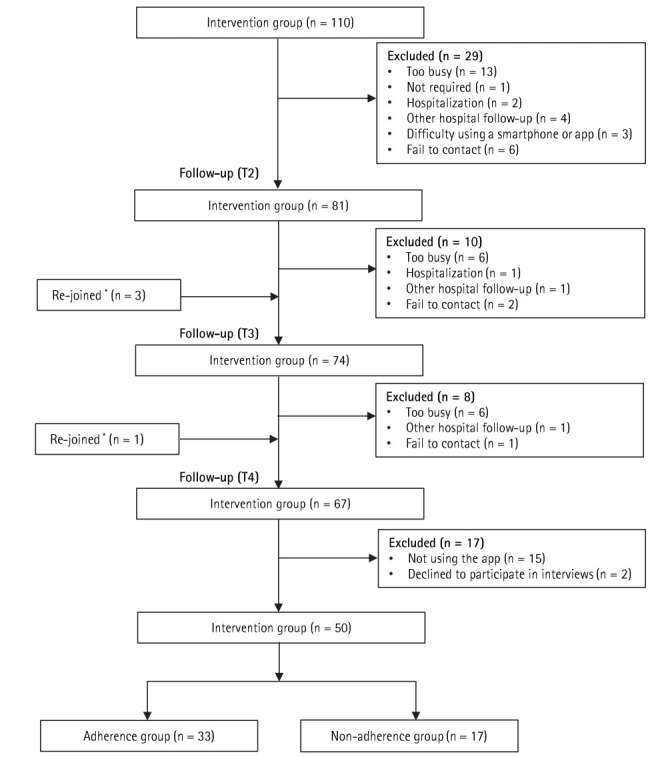
Flowchart of participant selection. T2 = 6-month follow-up; T3 = 12-month follow-up; T4 = 18-month follow-up. *Participants rejoined after missing the previous data collection.

Among participants enrolled in the parent study [5], those in the intervention group who completed the 18-month follow-up were included. Of the 110 individuals initially assigned to the intervention group, 67 completed the 18-month follow-up period. After excluding 15 participants who did not use the app and two who declined to participate in qualitative interviews, 50 participants were included in the final analysis (Figure 1). Owing to scheduling constraints, 25 participants were interviewed by telephone, consistent with previous research demonstrating that telephone interviews yield results comparable to face-to-face interviews [14]. Following Payne et al. [7], who observed that app-based dietary self-monitoring at least four times per week was significantly associated with short-term weight loss, participants were classified into two groups: the AG (n = 33), who used the app four or more times per week, and the NAG (n = 17), who used it less frequently.

### 3. Instruments

#### 1) Interview questions for qualitative data collection

The researchers tailored the interview questions for the AG and NAG to explore their distinct experiences with the app. Standardized questions included the following: “Can you describe your experience with the PSC app?”, “What efforts have you made to use the app?”, “How do you feel when using it regularly or not using it?”, “What challenges prevent regular use?” “What support would help you use the app more consistently?”. Follow-up questions such as “Why do you think that?” or “Could you explain more?” were used to elicit deeper responses. All interviews were audio-recorded, transcribed verbatim, and thoroughly reviewed to identify key statements.

#### 2) Quantitative data collection instruments

##### (1) General and disease-related characteristics

General and disease-related characteristics of the participants were collected using a structured questionnaire and medical record review. Participant characteristics included date of birth, sex, education level, regular exercise, physical activity, and occupation. Disease-related characteristics included BMI, medications, and diabetic complications.

##### (2) Self-care self-efficacy

Self-care self-efficacy was assessed using the Diabetes Management Self-Efficacy Scale (DMSES), originally developed by Bijl et al. [15] and translated into Korean by Lee et al. [16]. Permission was obtained from both the original developers and the translators. The 16-item scale evaluates self-efficacy in dietary management, blood glucose control, weight management, physical activity, and medical management. Responses were rated on a 10-point Likert scale, with higher scores indicating greater self-efficacy. The original DMSES reported a Cronbach’s α of .81, while the Korean version demonstrated .92. In this study, Cronbach's α was .91.

##### (3) Diabetes self-management

In this study, diabetes self-management were conceptualized as comprising three primary components: (1) diabetes self-care activities—including general diet, specific diet, exercise, blood glucose testing, foot care, and smoking—(2) physical activity, and (3) dietary intake. Diabetes self-care activities were measured using the Korean-translated version of the revised Summary of Diabetes Self-care Activities (SDSCA), developed by Toobert, Hampson, and Glasgow [17], with permission obtained from the authors. The SDSCA consists of 11 items across six domains: general diet, specific diet, exercise, blood glucose testing, foot care, and smoking. Smoking was assessed separately using binary (yes/no) responses and cigarette consumption, where applicable. The remaining items—related to the five self-care domains, excluding smoking—were rated on an 8-point Likert scale ranging from 0 to 7, reflecting the number of days the activity was performed in the past week. For each of these five domains, mean scores were calculated by averaging the responses to the relevant items. Higher scores indicated greater engagement in self-care activities. The average inter-item correlation across subscales was .47, and the test–retest reliability over 3~4 months was .40 [17]. Cronbach’s α was .66 in a study by Choi et al. [18] and .78 in the present study.

Physical activity was measured using the International Physical Activity Questionnaire (IPAQ) short form, Korean-translated [19]. The IPAQ evaluates vigorous (8.0 metabolic equivalents [METs]), moderate (4.0 METs), and walking activities (3.3 METs) over the past 7 days. Its reliability was supported by test–retest correlation coefficients ranging from *ρ* = 0.43 to 0.65, and validity was confirmed by a correlation coefficient of *r* = .27 when compared to accelerometer data [19].

Dietary intakes were assessed using the 24-hour recall method over three days, beginning the day before their outpatient visit and the two consecutive days. Entries were reviewed and follow-up calls addressed missing details. The three-day dietary intake by food group was analyzed using the Computer Aided Nutrition Analysis Program 4.0 (CAN Pro 4.0; The Korean Nutrition Society, Seoul, Korea).

##### (4) HbA1c

HbA1c levels were obtained from hospital medical records within 3 months before or after the survey. All samples were analyzed through blood tests conducted after a minimum fasting period of 4 hours.

### 4. Intervention

The PSC program, developed by Park, Lee, and Khang [20], is a self-regulation theory–based mobile intervention comprising four components: goal setting, education, monitoring, and feedback. Participants received personalized goals for physical activity, diet, medication adherence, blood glucose monitoring, smoking cessation, and foot care; exercise targets began at 50 minutes per session, seven times weekly, with 10.0% weekly increments up to 120 minutes, and diet goals were automatically adjusted each week based on achievement rates for each food group. Medication adherence was targeted at 100.0%, blood glucose monitoring at ≥ 1/day, smoking cessation for smokers, and foot care ≥ 5/week. Educational materials, consisting of brief 5-min videos and cartoon-based content, were provided through the mobile app. Participants were encouraged to view at least one educational material per week, and facilitators verified participants’ understanding by discussing key learning points during weekly check-ins. Participants recorded daily data on exercise (type/intensity, duration), diet (food type and portion size), medication adherence, and blood glucose levels using their glucometer. Smoking status and foot care were monitored weekly. Automated feedback messages were sent every morning at 8 a.m., summarizing the previous day’s best- and least-achieved behaviors. The app’s chatbot also provided tailored self-management strategies. Incentives were awarded as mileage points for completing goals, accessing educational content, and reviewing feedback, which were converted into gift certificates every 6 months. Facilitators provided monthly support via phone or text to monitor app usage and deliver motivational feedback.

### 5. Data collection

For the parent study [5], baseline data were collected between April 26, 2021, and March 31, 2022, with follow-ups every 6 months over 18 months. Data were collected at the Department of Endocrinology, Pusan National University Hospital, Yangsan-si, Republic of Korea. In the present study, baseline and 18-month follow-up data were analyzed to evaluate the effectiveness of the mobile app based on participants’ adherence levels. Following quantitative data collection, one-on-one interviews were conducted with participants who provided consent. Each interview lasted 15~20 min and was conducted in a private room within the outpatient endocrinology department.

### 6. Data Analysis

#### 1) Qualitative data analysis

This study employed qualitative content analysis, as described by Elo and Kyngäs [21], to identify overarching themes. Key sentences were highlighted, abstracted, and organized into thematic categories. The primary analysis was conducted by one researcher (DL), who prepared for qualitative data analysis by enhancing her expertise through doctoral coursework, attending qualitative research workshops, and conducting an extensive literature review. To ensure the validity of the findings, the extracted themes were reviewed and validated by an experienced nursing faculty member (HL), who provided critical feedback on the final thematic structure. Lincoln and Guba’s criteria for trustworthiness were applied to ensure rigor in the qualitative research process. To establish credibility, purposive sampling was used to recruit participants from the intervention group who completed the 18-month follow-up [5]. To enhance transferability, the findings were contextualized to allow their application to individuals with similar experiences, and relevant literature was reviewed to support the interpretation of adherence-related themes. Dependability was addressed by ensuring consistency and reliability through detailed documentation of participant selection, interview protocols, and analytical procedures. To ensure confirmability, participants’ statements were presented verbatim to allow readers to assess the accuracy and neutrality of the analysis and interpretation. The researchers also maintained objectivity and engaged in reflexivity to minimize bias throughout the research process.

#### 2) Quantitative data analysis

Quantitative analyses were conducted using SPSS version 23.0 (IBM Corp, Armonk, NY, USA), with two-tailed tests at a significance level of *p* < .05. Descriptive statistics, including means, standard deviations, frequencies, and percentages, were calculated to summarize participant characteristics. The Kolmogorov–Smirnov test was used to assess normality. To evaluate baseline homogeneity and between-group differences in outcome variables, independent t-tests were applied for normally distributed data, whereas the Mann–Whitney U test was used for non-normally distributed data. For within-group comparisons, paired t-tests were used for normally distributed data, whereas the Wilcoxon signed-rank test was used for non-normally distributed data. For categorical variables, between-group differences in proportions were analyzed using the chi-square test. When the expected cell counts were less than five, Fisher’s exact test was applied. McNemar’s test was used to assess within-group changes over time.

### 7. Ethical considerations

The parent study was approved by the Institutional Review Board (IRB) of the Pusan National University Hospital, Yangsan-si, Republic of Korea (IRB No. 05-2021-030). This study included only individuals who voluntarily consented to participate in qualitative interviews 18 month after the pretest. For the parent study, after receiving a detailed explanation of the study’s purpose and procedures, participants provided informed consent. The consent form contained comprehensive information regarding the study objectives, participant anonymity and confidentiality, and the right to refuse or withdraw from the study at any time. It also explicitly stated that participants could withdraw from the study at any time if they wished. Furthermore, participants were informed that all collected data would be used solely for research purposes, that their responses would remain anonymous, and that personal information would be stored in encrypted files on a password-protected computer for three years before being permanently destroyed.

## RESULTS

### 1. Baseline characteristics and group comparability

Fifty participants from the intervention group completed qualitative interviews. 33 in the AG and 17 in the NAG. Table 1 summarizes the baseline characteristics and homogeneity tests between the two groups. Overall, demographic and clinical characteristics of the groups were comparable at baseline and at outcome measures, except for metformin use, self-care self-efficacy, general diet, and smoking status. Specifically, a higher number of participants in the NAG used metformin (n = 10) compared with the AG (n = 8). Participants in the AG exhibited higher self-care self-efficacy scores (121.73 ± 22.74) than those in the NAG (102.12 ± 21.32). In the domain of diabetes self-care activities, the AG group also reported higher scores for general diet (3.83 ± 1.74) compared with the NAG group (2.68 ± 1.92). Regarding smoking status, a greater number of participants in the NAG were smokers (n = 9), whereas the majority of nonsmokers (n = 28) were in the AG.

### 2. Qualitative results

#### 1) Experiences of the participants in the AG

Qualitative analysis of interview data from 33 participants in the AG yielded 105 meaningful statements. These were categorized into eight subthemes, which were further integrated into four main themes (Table 2).

#### 2) Experiences of the participants in the NAG

Qualitative analysis of interview data from 17 NAG participants yielded 105 meaningful statements. These were categorized into eight subthemes, which were further integrated into four main themes (Table 3).

### 3. Quantitative results

#### 1) Effects of the PSC app on self-care self-efficacy, diabetes self-care activities, and HbA1c

Among the subdomains of diabetes self-care activities, foot care showed significantly greater improvement in the AG than in the NAG, both in between- and within-group comparisons. Over time, participants in the AG demonstrated significant improvements in general diet, whereas those in the NAG showed improvements in exercise. Additionally, HbA1c levels significantly decreased in the AG, whereas no significant changes were observed in the NAG during the same period (Table 4).

## DISCUSSION

This study examined the experiences of individuals using the PSC program and its effects on self-care self-efficacy, diabetes self-care activities, physical activities, dietary intakes and HbA1c levels according to adherence levels among adults with T2D. The findings demonstrated significant improvements over time in the general diet and foot care subdomains of diabetes self-care activities, as well as in HbA1c levels in the AG. These results suggest that the PSC program effectively enhanced dietary habits, foot care practices, and glycemic control.

Previous research [5] demonstrated that the intervention was effective in improving HbA1c levels among participants in the intervention group. However, in the present study, significant improvements were observed only in the adherence group, suggesting that the intervention’s effectiveness may be influenced by differences in participant characteristics between the adherence and non-adherence groups. The NAG exhibited higher rates of metformin use and smoking and lower levels of self-efficacy and general diet adherence compared to the AG. Qualitative interviews revealed that NAG participants experienced challenges in diabetes self-management due to low motivation, decreased willpower, and the burden of daily life. Consequently, no significant improvement in HbA1c was observed after the intervention, suggesting the need for early identification and tailored management of non-adherent individuals. Previous studies have reported that intervention effectiveness is strongly influenced by adherence, with greater adherence associated with larger reductions in HbA1c [6]. Self-efficacy is also positively correlated with self-care adherence among patients with T2D, and higher self-efficacy leads to better self-management behaviors [22]. Although facilitators in the PSC program provided motivational support through phone calls and text messages, these efforts appeared insufficient to improve adherence among non-adherent participants. Gilcharan Singh et al. [23] found that motivational interviewing significantly improved self-efficacy among overweight or obese patients with T2D receiving a structured intervention. Similarly, studies incorporating telephone-based motivational interviewing have demonstrated sustained improvements in self-efficacy and diabetes self-management at 3- and 6-month follow-ups. These findings suggest that integrating motivational interviewing via phone or text into future interventions may enhance adherence and promote continued engagement in diabetes self-care.

The main barriers among NAG were difficulties in using the mobile application and psychological and physical burden. These findings are consistent with previous studies reporting that technical issues and low user awareness are major obstacles to digital diabetes interventions [11]. Moreover, users’ perceived usefulness of mobile applications has been shown to influence not only intervention engagement but also clinical outcomes such as HbA1c [10,12]. Therefore, enhancing app usability to minimize technical barriers and improving users’ awareness of its benefits are essential for promoting adherence. For example, visual data presentation, intuitive navigation, image-based input functions (e.g., food photo uploads), and picture-oriented user interfaces can reduce user burden and facilitate engagement [12,24,25]. Moreover, to enhance the acceptability of mobile applications, it is essential to improve their usability and perceived usefulness, and thereby develop concrete strategies to boost users’ awareness and sustained engagement [26].

Improvements in dietary behavior were observed only in the AG, where family support—particularly from spouses—played a pivotal role. In contrast, NAG participants frequently lacked such support, leading to lower engagement in dietary self-care. Prior studies [27,28] have similarly demonstrated a strong positive association between family support and diabetes self-management, indicating that inclusion of family members in educational and monitoring components may enhance intervention effectiveness. However, in Zeren et al. [27], family support scores were significantly lower among those whose expenditures exceeded their income or who were unemployed, suggesting that economic vulnerability may limit family support capacity. In this light, policy interventions such as financial assistance for low-income households and expanded home-based nursing services are warranted. Such structural supports could enable family members to more actively participate in care, thereby potentially boosting intervention uptake among non-adherent individuals.

In the parent study [5], participants in the intervention group showed a significant improvement in HbA1c compared to those in the control group. However, in the present study, this improvement was observed only among adherent participants. Qualitative findings revealed that participants in the adherence group reported an increased awareness of the importance of diabetes self-management and a heightened sense of responsibility in their daily routines after participating in the PSC program. They also stated that regular feedback and consistent monitoring through the mobile application served as key motivational factors for sustaining self-management behaviors. Similar results have been consistently reported in previous studies. For instance, Poppe et al. [29] demonstrated that weekly goal setting, self-monitoring, and tailored feedback effectively reduced sedentary time and increased physical activity. Likewise, Sze et al. [30] found that personalized step-count goals and strategies to overcome barriers contributed to improvements in physical activity, BMI, and HbA1c levels. Also, Marques et al. [31] reported that app-based foot care education combined with phone feedback improved foot care scores and frequency. These findings suggest that integrating mobile app education with continuous feedback is an effective strategy for improving diabetes self-management and preventing complications. Collectively, these findings underscore that self-regulation theory–based interventions using mobile applications that incorporate education, monitoring, individualized goal setting, and feedback play a pivotal role in enhancing diabetes self-management.

This study applied the PSC program to adults with T2D using a mixed-methods design to explore participants’ experiences based on adherence. The significance of this study lies in its analysis of the benefits, challenges, and barriers to diabetes management via a mobile app, thereby enhancing our understanding of mobile health–assisted diabetes care. Self-monitoring, positive feedback, education, and personalized goal setting have been shown to improve diabetes management behaviors effectively. Furthermore, the findings underscore the importance of family support in sustaining long-term diabetes management. Both the AG and NAG participants provided recommendations for improving the mobile app, suggesting the need for continued development to enhance user experience and engagement. However, this study has certain limitations. First, the declining sample size over time may limit the generalizability of the findings. Second, self-efficacy and self-care were assessed using self-report questionnaires, which may not fully reflect actual behaviors, suggesting the need for more objective tools. Third, app improvements—such as expanding the food database and enhancing reminder functions—are recommended. Finally, interviews conducted after 18 months may have been influenced by recall bias, potentially affecting the accuracy of participants’ reports.

## CONCLUSION

This mixed-methods study evaluated the effectiveness of the PSC program in adults with T2D. Participants in the AG demonstrated significant improvements in diet, foot care, and HbA1c levels, supported by app-based feedback, family involvement, and facilitator guidance. In contrast, NAG participants reported limited social support, awareness, and reduced motivation. These findings highlight the need for developing targeted strategies to strengthen engagement and adherence in less responsive groups.

## Figures and Tables

**Table 1.** Characteristics of Study Participants (N = 50)

Characteristics	Total	AG (n = 33)	NAG (n = 17)	t or x^2^	*p*
Age (years)	55.80 ± 8.03	55.94 ± 8.81	55.53 ± 6.48	0.15	.883
40∼49	11 (22.0)	7 (21.2)	4 (23.5)	0.15	1.000^†^
50∼59	21 (42.0)	14 (42.4)	7 (41.2)		
≥ 60	18 (36.0)	12 (36.4)	6 (35.3)		
Sex					
Men	26 (52.0)	15 (45.5)	11 (64.7)	1.67	.197
Women	24 (48.0)	18 (54.5)	6 (35.3)		
Education					
≤ Middle school	6 (12.0)	5 (15.2)	1 (5.9)	-	.650
≥ High school	44 (88.0)	28 (84.8)	16 (94.1)		
Employed					
Yes	34 (68.0)	22 (66.7)	12 (70.6)	0.08	.778
No	16 (32.0)	11 (33.3)	5 (29.4)		
Regular exercise					
Yes	24 (48.0)	19 (57.6)	5 (29.4)	3.57	.059
No	26 (52.0)	14 (42.4)	12 (70.6)		
BMI (kg/m^2^)	25.85 ± 3.87	25.77 ± 4.11	26.00 ± 3.49	−0.19	.847
18.5∼22.9	13 (26.0)	9 (27.3)	4 (23.5)	0.18	1.000^†^
23.0∼24.9	9 (18.0)	6 (18.2)	3 (17.6)		
≥ 25.0	28 (56.0)	18 (54.5)	10 (58.9)		
Diabetes medication use					
Metformin	18 (36.0)	8 (24.4)	10 (58.8)	-	.028^†^
Sulfonylureas	20 (40.0)	11 (33.3)	9 (52.9)	-	.229^†^
Insulin	15 (30.0)	11 (33.3)	4 (23.5)	-	.533^†^
SGLT2	13 (26.0)	7 (21.2)	6 (35.3)	-	.322^†^
Others^∮^	35 (70.0)	24 (72.7)	11 (64.7)	-	.746^†^
Diabetic complications					
Yes	10 (20.0)	8 (24.2)	2 (11.8)		
Number of complications					
1	8 (80.0)	6 (75.0)	2 (100.0)	-	.429^†^
≥ 2	2 (20.0)	2 (25.0)	0 (0.0)		
Self-care self-efficacy	115.06 ± 23.96	121.73 ± 22.74	102.12 ± 21.32	2.95	.005
Diabetes self-management					
Diabetes self-care activities	3.29 ± 1.39	3.49 ± 1.41	2.89 ± 1.31	1.47	.147
General diet	3.44 ±1.87	3.83 ± 1.74	2.68 ± 1.92	−2.15	.037
Specific diet	4.27 ± 1.26	4.45 ± 1.20	3.91 ± 1.33	−1.46	.150
Exercise	3.00 ± 2.20	3.29 ± 2.18	2.44 ± 2.20	−1.30	.200
Blood glucose test	3.88 ± 2.32	4.02 ± 2.49	3.62 ± 2.00	−0.56^‡^	.576
Foot care	2.73 ± 2.57	2.74 ± 2.62	2.71 ± 2.55	−0.07^‡^	.941
Smoking					
Yes	14 (28.0)	5 (15.2)	9 (52.9)	-	.008^†^
No	36 (72.0)	28 (84.8)	8 (47.1)		
Physical activity (METs) (n = 47)	1305.50 ± 1283.54	1429.97 ± 1158.79	1063.88 ± 1505.25	−0.96	.345
Inactive	17 (36.2)	9 (29.0)	8 (50.0)	2.61	.301^†^
MA	25 (53.2)	19 (61.3)	6 (37.5)		
HEPA	5 (10.6)	3 (9.7)	2 (12.5)		
Dietary intake (AIR, %)					
Grains	156.00 ±50.39	159.12 ± 49.14	149.93 ± 53.73	0.61	.547
Protein foods	141.58 ±59.67	148.40 ± 63.33	128.34 ± 50.99	1.13	.264
Vegetables	60.83 ± 41.76	64.94 ± 45.88	52.86 ± 32.10	−1.18^‡^	.239
Fruits	34.81 ± 44.23	36.95 ± 51.62	30.64 ± 25.16	−0.67^‡^	.504
Dairy	22.24 ± 32.41	20.44 ± 28.65	25.73 ± 39.45	−0.09^‡^	.925
HbAlc (%)	7.96 ± 1.33	8.01 ± 1.52	7.86 ± 0.89	−0.31^‡^	.758

Values are presented as the mean ± standard deviation or n (%). AG = Adherence group; NAG = Non-adherence group; BMI = Body max index; SGLT2 = Sodium-glucose cotransporter 2; METs = Metabolic equivalents; MA = Minimally active; HEPA = Health-enhancing physical activity; AIR = Percentage of actual 3-day average intake/recommended foods intake; HbA1c = Hemoglobin A1c. ^†^Fisher’s exact test; ^‡^Mann–Whitney U test;^∮^Includes combination drugs containing two or more active ingredients, DPP-4 inhibitors, GLP-1 receptor agonists, and thiazolidinediones.

**Table 2.** Qualitative Themes and Sub-themes in the Adherence Group (N = 33)

Themes clusters	Themes	Statement
Living a healthy life with a diabetes management app	Increased responsibility and routine self-management	By regularly logging everything, I felt my blood sugar levels improved, I became more consistent with exercise, and I started being more mindful about my diet overall. (AG 96)
	Recognizing self-management progress	I used to drink mixed coffee, but now I switch to black coffee whenever I want some. (AG 117)
Commitment to consistent self-management	Engaging in regular physical activity	I usually walked near my house for about 40 to 50 minutes. Although I missed a couple of days when it was too cold or during the summer heat, I tried to walk almost every day. (AG 164)
	Psychological and motivational challenges	It’s just an excuse, saying I’m too busy. Honestly, it only takes a moment to log into the app, but I feel like I should do it after checking my blood sugar. Yet, because I say I’m busy, I end up skipping the blood sugar check altogether. (AG 102)
User experience and suggestions for app improvement	Desire for more user-friendly features	I wish the app had alarms, like ‘check your blood sugar' or 'take your medication. (AG 64)
	Improved dietary tracking functionality	I wish the app were simpler. For example, I could just enter 'two bowls of rice,' but instead, I have to search for white rice or barley rice, and sometimes the exact type I ate isn’t even there. (AG 102)
Support and collaboration for effective self-management	Family and facilitator support	My wife, family, and others who care about my health often remind me not to eat certain foods, which helps a lot. (AG 142)
	Motivation through feedback and regular check-ins	I changed my mindset a little bit by increasing the number of workouts and stuff, even though it's a little bit of a hassle, and decreasing the amount of food a little bit. (AG 45)

AG = Adherence group.

**Table 3.** Table 3. Qualitative Themes and Sub-themes in the Non-adherence Group (N = 17)

Themes clusters	Themes	Statement
The role of support in diabetes self-management	Importance of support for motivation	I'd get feedback via text or phone, and I'd realize I needed to exercise a little bit more or tweak my diet a little bit. (NAG 38)
	Lack of social support	If patients share statements like 'I do this' or 'I exercise' with each other, it could motivate them to manage their diabetes. (NAG 188)
Barriers to effective diabetes management	Challenges with the mobile app	I wish the mobile app made data entry easier, especially since I'm so busy. It feels overly detailed to me. While experts might think it’s not too complex, for beginners like us, it can be a bit tedious. (NAG 38)
	Physical and emotional barriers	My glycated hemoglobin is high, and I know I should manage my diabetes better, but everything feels overwhelming. I just feel unwell and want to lie down. (NAG 188)
Recognition of the need for better self-management	Growing awareness and behavior change	Because you take care of me, I don’t drink alcohol or eat sweets anymore, just a little bit. I used to like meat, but now I’m a vegetarian. (NAG 66)
	Motivation through app features	You took a quiz, made a guess, and received a gift certificate for answering correctly. It was fun. (NAG 39)
Psychological responses to diabetes management	Emotional struggles and stress	I didn’t log my diabetes data properly, so I felt sorry when I got a call and thought I should try again. (NAG 33)
	Positive reinforcement and confidence	I initially checked my diabetes on the app out of habit, but over time, it motivated me to exercise, so I started incorporating some workouts as well. (NAG 39)

NAG = Non-adherence group.

**Table 4.** Effects of the Program on Diabetes Self-care Self-efficacy, Self-management, and HbA1c (N = 50)

Variables		T1	T4	t or Z^†^ (*p*)	Mean differences (T4−T1)		
						t or Z^‡^	*p*
Self-care self-efficacy	AG (n = 33)	121.73 ± 22.74	117.00 ± 20.54	1.10 (.278)	−4.73 ± 24.61	1.43	.159
	NAG (n = 17)	102.12 ± 21.32	108.53 ± 23.16	−0.92 (.372)	6.41 ± 28.76		
Diabetes self-management							
Diabetes self-care activities	AG	3.49 ± 1.41	4.33 ± 1.31	−3.79 (.001)	0.84 ± 1.27	−0.99	.326
	NAG	2.89 ± 1.31	3.38 ± 1.26	−2.23 (.041)	0.49 ± 0.92		
General diet	AG	3.83 ± 1.74	4.64 ± 1.65	−2.68 (.012)	0.80 ± 1.72	−0.49^‡^	.621
	NAG	2.68 ± 1.92	3.32 ± 1.13	−1.30^†^ (.194)	0.65 ± 1.77		
Specific diet	AG	4.45 ± 1.20	4.39 ± 1.07	−0.32^†^ (.752)	−0.06 ± 1.13	−1.04^‡^	.297
	NAG	3.91 ± 1.33	4.06 ± 1.29	−0.39 (.699)	0.15 ± 1.54		
Exercise	AG	3.29 ± 2.18	3.74 ± 1.95	−1.26 (.218)	0.45 ± 2.08	−0.28^‡^	.780
	NAG	2.44 ± 2.20	3.06 ± 1.98	−2.35 (.032)	0.62 ± 1.08		
Blood glucose test	AG	4.02 ± 2.49	4.83 ± 2.23	−1.51^†^ (.131)	0.82 ± 2.70	−0.84^‡^	.404
	NAG	3.62 ± 2.00	3.68 ± 2.72	−0.11 (.915)	0.06 ± 2.23		
Foot care	AG	2.74 ± 2.62	4.62 ± 2.37	−3.98^†^ (< .001)	1.88 ± 2.15	−2.22	.031
	NAG	2.71 ± 2.55	3.24 ± 2.21	−1.20^†^ (.230)	0.53 ± 1.78		
Smoking							
Yes	AG	5 (10.0)	6 (12.0)	-	-	-	-
	NAG	9 (18.0)	7 (14.0)	-	-		
No	AG	28 (56.0)	27 (54.0)	-	-	-	-
	NAG	8 (16.0)	10 (20.0)	-	-		
Physical activity (METs)	AG	1429.97 ± 1158.79	2364.70 ± 1695.72	−3.30^†^ (.001)	934.73 ± 1353.72	1.02	.321
	NAG	1063.88 ± 1505.25	2645.76 ± 2252.52	−2.43^†^ (.015)	1581.88 ± 2441.20		
Dietary intake (AIR, %)							
Grains intake	AG	159.12 ± 49.14	140.98 ± 54.90	1.41 (.169)	−18.14 ± 74.00	0.33	.745
	NAG	149.93 ± 53.73	142.72 ± 44.79	0.58 (.572)	−10.74 ± 74.34		
Protein foods intake	AG	148.40 ± 63.33	140.30 ± 88.74	0.51 (.616)	−8.10 ± 92.02	1.14	.260
	NAG	128.34 ± 51.00	157.36 ± 92.75	−1.05 (.309)	23.54 ± 89.35		
Vegetable intake	AG	64.94 ± 45.88	66.77 ± 41.19	−0.21^†^ (.837)	1.83 ± 46.01	−0.53^†^	.594
	NAG	52.86 ± 32.10	59.05 ± 40.45	−0.83 (.408)	3.73 ± 44.98		
Dairy intake	AG	20.44 ± 28.65	22.54 ± 22.14	−0.51^†^ (.611)	2.10 ± 34.01	−0.71^‡^	.481
	NAG	25.73 ± 39.45	14.93 ± 15.91	−0.31^†^ (.753)	−11.09 ± 38.14		
Fruits intake	AG	36.95 ± 51.62	47.02 ± 38.07	−1.33^†^ (.183)	10.07 ± 44.73	0.56	.580
	NAG	30.65 ± 25.16	49.41 ± 36.31	−2.05 (.041)	16.92 ± 29.11		
HbA1c (%)	AG	8.01 ± 1.52	7.47 ± 0.98	−2.93^†^ (.003)	−0.76 ± 1.56	−0.23^‡^	.817
	NAG	7.86 ± 0.89	7.59 ± 0.71	−1.61^†^ (.108)	−0.50 ± 1.06		

Values are presented as the mean ± standard deviation or n (%). HbA1c=Hemoglobin A1c; T = Time; T1 = Baseline; T4 = 18-Month follow-up; AG = Adherence group; NAG = Non-adherence group; METs = Metabolic equivalents; AIR = Percentage of actual 3-day average intake/recommended foods intake. ^†^Wilcoxon signed-rank test; ^‡^Mann-Whitney U test.

## Data Availability

The data that support the findings of this study are available from the corresponding author upon reasonable request.
